# Supplementation during pregnancy with small‐quantity lipid‐based nutrient supplements or multiple micronutrients, compared with iron and folic acid, increases women's urinary iodine concentration in semiurban Ghana: A randomized controlled trial

**DOI:** 10.1111/mcn.12570

**Published:** 2017-12-06

**Authors:** Seth Adu‐Afarwuah, Rebecca T. Young, Anna Lartey, Harriet Okronipa, Per Ashorn, Ulla Ashorn, Mamane Zeilani, Kathryn G. Dewey

**Affiliations:** ^1^ Department of Nutrition and Food Science University of Ghana Accra Ghana; ^2^ Program in International and Community Nutrition, Department of Nutrition University of California Davis California USA; ^3^ Centre for Child Health Research University of Tampere Faculty of Medicine and Life Sciences, and Tampere University Hospital, Department of Pediatrics Tampere Finland; ^4^ Nutriset S.A.S. Malaunay France

**Keywords:** iLiNS DYAD‐Ghana, iodine intakes, multiple micronutrient capsules, pregnant women, small‐quantity lipid‐based nutrient supplements, urinary iodine concentration

## Abstract

There is little information on whether prenatal multiple micronutrient (MMN) supplements containing iodine affect women's iodine status. In the International Lipid‐based Nutrient Supplements DYAD‐Ghana trial, we aimed to assess women's urinary iodine concentration (UIC, μg/L) during pregnancy, as one of the planned secondary outcomes. Women (*n* = 1,320) <20 weeks of gestation were randomized to consume 60 mg iron and 400 μg folic acid per day (iron and folic acid [IFA]); 18 vitamins and minerals including 250 μg iodine per day (MMN); or 20 g/day of small‐quantity lipid‐based nutrient supplements (LNS) with the same and additional 4 vitamins and minerals as the MMN (LNS). In a subsample (*n* = 295), we tested differences in groups' geometric mean UICs at 36 weeks of gestation controlling for baseline UIC and compared the geometric means (approximately median UICs) with the World Health Organization (WHO) cut‐offs: median UIC <150, 150–249, and ≥500 reflecting low, adequate, and excessive iodine intakes, respectively. At baseline, overall median UIC was 137. At 36 weeks of gestation, controlling for baseline UIC, geometric mean (95% confidence interval) UICs of the MMN (161 [133, 184]) and LNS (158 [132, 185]) groups did not differ; both values were significantly greater (overall *p* = .004) than that of the IFA group (116 [101, 135]). The median UICs of the MMN and LNS groups were within the WHO “adequate” range, whereas that of the IFA group was below the WHO adequate range. In this setting, supplementation during pregnancy with small‐quantity LNS or MMN providing iodine at the WHO‐recommended dose, compared with IFA, increases the likelihood of adequate iodine status.

**Abbreviations**
IDD: iodine deficiency disorders
IFA: iron and folic acid
LNS: lipid‐based nutrient supplement
MMN: multiple micronutrients
SQ‐LNS: small‐quantity lipid‐based nutrient supplement
UIC: urinary iodine concentration

## 1 INTRODUCTION

Iodine is needed for the synthesis of thyroid hormones; these hormones are required for body metabolism and are key in the development of the central nervous system in fetuses and children (de Escobar, Obregon, & del Rey, 2007). Consequences of iodine deficiency disorders (IDD) among women include miscarriage, impaired mental function, development of goitre, and delivery of stillborn or neurocognitively impaired infants (United Nations Children's Fund [UNICEF], 2007; World Health Organization [WHO], 2007).

In 1994, universal salt iodization was recommended as the main strategy for preventing iodine deficiency (WHO, 1994; WHO, 2007), and iodized salt consumption has increased since then (Tran, Hetzel, & Fisher, 2016) due to national salt iodization programmes (UNICEF, 2008; WHO, 2007). Nonetheless, in 2013, 35% of the global population was at risk of iodine deficiency because of inadequate intakes (WHO, 2013), and in Africa, 11 countries remained iodine deficient (Jooste, Andersson, & Assey, 2013). Thus, despite the substantial progress in the prevention of IDD, iodine deficiency continues to be a public health problem in many countries (Tran et al., 2016; UNICEF, 2007).

In 2007, the WHO increased the then Food and Agriculture Organization of the United Nations recommended nutrient intake for iodine during pregnancy (Food and Agriculture Organization of the United Nations/WHO, 2005) from 200 to 250 μg/day (WHO Secretariat et al., 2007) and called for possible supplementation or food fortification with iodine for pregnant women in areas where universal salt iodization is not fully implemented and/or pregnant women might not be adequately covered by iodized salt in order to ensure optimal brain development of their infants (WHO/UNICEF, 2007).

The national salt iodization programme in Ghana began more than two decades ago (Government of Ghana, 1996). According to the 2014 Ghana Demographic and Health Survey (Ghana Statistical Service [GSS] et al., 2015), only 39% of households in Ghana have adequately iodized salt (15+ ppm). Along with previous reports (Jooste et al., 2013; UNICEF, 2008), this estimate suggests that many pregnant women in the country may not consume adequate iodine. Because deficiencies of micronutrients often occur together and not in isolation (Bailey, West, & Black, 2015; Haider & Bhutta, 2017; UNICEF/WHO/United Nations University, 1999), it is possible that pregnant women in Ghana might benefit from the provision of a multiple micronutrient (MMN) regimen that includes iodine, as compared with the provision of iodine alone.

In the International Lipid‐based Nutrient Supplements (iLiNS) DYAD‐Ghana trial, as part of the iLiNS Project, we tested the impact of prenatal consumption of small‐quantity lipid‐based nutrient supplements (SQ‐LNS; Arimond et al., 2015) or MMN capsules on various maternal (Adu‐Afarwuah, Lartey, Okronipa, Ashorn, Zeilani, et al., 2016; Adu‐Afarwuah et al., 2017) and child (Adu‐Afarwuah et al., 2015; Adu‐Afarwuah, Lartey, Okronipa, Ashorn, Peerson, et al., 2016) outcomes. Only a few such studies (Haider & Bhutta, 2017) comparing the impact of MMN supplementation versus iron and folic acid [IFA] supplementation during pregnancy have reported on women's urinary iodine concentration (UIC), even though the elimination of iodine deficiency is an international priority (UNICEF, 2008). In the analyses reported herein, we aimed to compare the UIC among the three groups of pregnant women enrolled in the iLiNS DYAD‐Ghana trial (UIC was one of the planned secondary outcomes of the trial) and to assess the groups' iodine intake adequacy on the basis of the WHO cut‐offs for UIC.

**Key messages**

- In this semiurban setting in Ghana, the median spot urinary iodine concentration of pregnant women at <20 weeks of gestation (137 μg/L) was below the World Health Organization range (150–249 μg/L) for adequate iodine intake. Only about one third of the women reportedly used iodized salt *usually*, or *always*, despite a national salt iodization programme.
- During pregnancy, daily supplementation with multiple micronutrients or small‐quantity lipid‐based nutrient supplements, compared with iron and folic acid, increased the likelihood of adequate iodine status.
- Regular monitoring of the iodine status of pregnant women is needed to ensure the elimination of iodine deficiency in Ghana.

## 2 METHODS

### 2.1 Study setting, design, and participants

The setting, design, and participants of the iLiNS DYAD‐Ghana trial have been described elsewhere (Adu‐Afarwuah et al., 2015). Briefly, we conducted the study in the semiurban Somanya‐Odumase‐Kpong area, about 70 km north of Accra, Ghana. The study was designed as a partially double‐blind, parallel, individually randomized, controlled trial with three equal‐size groups and was approved by the ethics committees of the University of California, Davis; the Ghana Health Service; and the University of Ghana Noguchi Memorial Institute for Medical Research and registered at http://clinicaltrials.gov (Identifier NCT00970866).

Between December 2009 and December 2011, pregnant women attending usual antenatal clinics in the four major health facilities in the area were screened if they were ≥18 years old; ≤20 weeks of gestation; and had fully completed antenatal cards. Women were excluded if any of the following applied: intention to move out of the area within the next 2 years; milk or peanut allergy; unwillingness to receive field workers or take study supplement; participation in another trial; gestational age >20 weeks before completion of enrolment; and antenatal card indicated HIV infection, asthma, epilepsy, tuberculosis, or malignant disease.

### 2.2 Group assignments and micronutrient supplements

As described previously (Adu‐Afarwuah et al., 2015), after baseline assessments, pregnant women were randomized to receive either 60 mg iron and 400 μg folic acid per day (hereafter, IFA supplement or group) or 18 vitamins and minerals (including 20 mg iron) per day (hereafter, MMN supplement or group) or 20 g/day of SQ‐LNS containing similar micronutrients as the MMN supplement, plus calcium, phosphorus, potassium, and magnesium, as well as energy (118 kcal/day) and macronutrients (e.g., protein and essential fatty acids; hereafter, SQ‐LNS or LNS group). The IFA and MMN were supplied by DSM South Africa in 10‐capsule‐blister packs and the SQ‐LNS by Nutriset S.A.S. (Malaunay, France) in individual 20‐g sachets. The nutrient contents of the supplements (Adu‐Afarwuah et al., 2015) and the rationale for the concentrations used (Arimond et al., 2015) have been published. For most of the vitamins and minerals in the MMN and SQ‐LNS, the contents were either 1*×* or 2*×* the recommended dietary allowance for pregnancy (Arimond et al., 2015), except for iron (Adu‐Afarwuah, Lartey, Okronipa, Ashorn, Zeilani, et al., 2016). We included 250 μg/day for iodine based on the WHO recommendation (WHO Secretariat et al., 2007).

The group assignments were performed as follows: the study statistician at UC Davis, Janet M. Peerson, used a computer‐generated (SAS version 9.4) scheme to develop the group allocations in blocks of 9, with the IFA and MMN groups coded by six different colours, to maintain blinding. In Ghana, someone unrelated to the randomization placed the group assignments in opaque envelopes, which were labelled with the block numbers, sealed, and stacked in increasing order of block numbers. The labelling was done in pencil on the side of the envelopes not conspicuous to the women, so as to not influence the women's choices. At each enrolment, the study nurse took the topmost nine envelopes off the stack, shuffled them, and asked the woman to pick one of the nine envelopes, which revealed the group assignment. The study nurse then returned the unused envelopes to the top of the stack. This process continued until all of the envelopes prepared for the target sample were used. When there were less than nine women left to be enrolled, it was no longer possible for a participant to pick one of the nine envelopes, except for the study nurse to shuffle whatever number of envelopes that remained, for the women to make a pick. At no point during randomization was it possible for the study nurse to guess the remaining allocations, because she had no knowledge of the randomization scheme. Any allocation information was kept securely by the field supervisor in Ghana and the study statistician at UC Davis only.

### 2.3 Intervention

The intervention procedures were reported previously (Adu‐Afarwuah et al., 2015; Adu‐Afarwuah, Lartey, Okronipa, Ashorn, Peerson, et al., 2016). At enrolment, the study nurse gave each woman a 2‐week supply of the assigned supplement and provided instructions on how to consume it (IFA and MMN, with water after a meal, one capsule per day; SQ‐LNS, mixed with any prepared food, one 20‐g sachet per day). Thereafter, field workers delivered fresh supplies of supplement and monitored supplement intakes and morbidity biweekly (Adu‐Afarwuah et al., 2015). Women were told not to consume more than one capsule (IFA and MMN groups) or sachet (LNS group) per day, even if they forgot to take the supplement the previous day or days. Women travelling out of the study area for periods beyond the next biweekly visit were given extra supplies of supplements for the period they intended to be away.

It was not possible to blind field workers and participants to women who received the capsules (IFA and MMN) versus those who received SQ‐LNS, because of the apparent differences between these supplements. However, the laboratory staff who collected or analysed the samples were blinded to the group assignments.

### 2.4 Procedures and outcome measures

At baseline, we collected data on women's demographic and socio‐economic characteristics and how often women used iodized salt (whether *never*, *sometimes*, *usually*, or *always*). We presumed that women knew iodized salt versus non‐iodized salt by the packaging and cost (even if they could not read any labels), because the latter is often not packaged and costs less. Further, we conducted anthropometric and laboratory assessments and obtained spot urine samples, which were frozen until analysed (Adu‐Afarwuah et al., 2015). We measured women's weight (Seca 874, Seca), height (Seca 217, Seca), mid‐upper arm circumference (Shorr tapes), and triceps skinfold thickness (Holtain calipers) by using standard procedures and determined blood haemoglobin concentration (HemoCue, HemoCue AG), malaria parasitaemia (Vision Biotech), and gestational age (mostly by ultrasound biometry, Aloka SSD 500). We calculated the assets index, housing index, and Household Food Insecurity Access Scale score as proxy indicators of background socio‐economic status, by using principal component analysis (Coates, Swindale, & Bilinsky, 2007). At 36 weeks of gestation, we asked the women again about their frequency of using iodized salt. The women returned to the laboratory (last laboratory visit before delivery), whereupon we repeated the collection of spot urine samples.

Urine samples were air‐freighted on dry ice to the laboratories of the Medical Research Council in Cape Town, South Africa, where UIC was determined. First, the urine samples were manually digested in a 96‐well plate by using ammonium persulfate. The digested samples were then transferred to a new microplate for the Sandell–Kolthoff reaction, and UIC was read at 405 nm (Henjum et al., 2016; Jooste & Strydom, 2010).

The secondary outcomes evaluated here were geometric mean UIC (μg/L) at 36 weeks of gestation and median change in UIC over the intervention period.

### 2.5 Sample size basis and data analysis

For women's UIC during pregnancy (as well as several other biochemical outcomes in our trial), we based the target sample size on detecting an effect size or Cohen's *d* (Cohen, 1988) of ≥0.5 between any two groups, with a two‐sided 5% test and 80% power. This required 105 women per group or 315 women for the three groups, after taking into account up to 25% attrition. Subsequently, we randomly selected a subsample of 315 women from the 810 women enrolled after we had corrected the mislabelling situation we reported previously (Adu‐Afarwuah et al., 2015; Adu‐Afarwuah, Lartey, Okronipa, Ashorn, Peerson, et al., 2016), to ensure that none of the women in the UIC analyses had been exposed to unintended supplements during pregnancy. At 36 weeks of gestation, we had UIC values for 292 women, which gave >93% power to detect an effect size of 0.5 between any two groups.

The analysis for the results presented here was part of the iLiNS DYAD‐Ghana statistical analysis plan, which was developed and posted at our website (http://www.ilins.org) before data analysis began. Both the statistical analysis plan and trial protocol at http://clinicaltrials.gov list women's UIC as a secondary outcome to be analysed separately. We analysed data on an intention‐to‐treat basis (by including women regardless of adherence to treatment) and by using SAS for Windows Release 9.4 (Cary, NC, USA). We summarized the background characteristics at enrolment, by group, as mean ± *SD* or frequency (%). Because UICs are known to be not normally distributed (WHO, 2013), we summarized baseline UIC and change in UIC from baseline to 36 weeks of gestation as median (Q1, Q3), by group, and compared UIC of groups at 36 weeks of gestation after natural log transformation.

The comparison at 36 weeks of gestation was performed by using analysis of covariance (SAS PROC GLIMMIX), with Tukey adjustment for multiple comparison. The prespecified variables evaluated as potential covariates and for interaction in bivariate models were gestational age at enrolment, years of formal education, parity (nulliparous or parous), season of enrolment (wet or not wet), household assets index (by principal component analysis), Household Food Insecurity Access Scale score (Coates et al., 2007), reported use of iodized salt, and baseline UIC. The covariate for use of iodized salt was created from the frequency of using iodized salt at baseline and 36 weeks of gestation by assigning 0, 1, 2, and 3 to *never*, *sometimes*, *usually*, and *always*, respectively, and averaging the coded responses across the two time points. Values of this covariate ranged from 0 to 3, where higher values represented higher frequency of use of iodized salt. Only the prespecified variables significantly associated with the outcome at <.1 level of significance were included as covariates. Interactions with *p*‐for‐interaction <.1 were considered significant.

We performed the analysis of covariance twice: first, with baseline UIC as the only covariate in the model and, second, with all of the identified covariates in the model. A visual inspection and a Shapiro–Wilk test of normality revealed that the residuals of the models were normally distributed. We obtained the group geometric means of UICs and their 95% confidence intervals by back‐transformation. The geometric mean UIC is an approximate estimator of median UIC (Hauschke, Steinijans, & Pigeot, 2007; Thomas, 1990). For prespecified variables with a significant interaction, we performed subgroup analyses to determine whether intervention groups differed in the different subgroups. Each interaction variable was considered separately in the model to avoid collinearity.

Finally, to assess the adequacy of iodine intakes of groups, we compared the group geometric mean (approximately the group median) UICs with WHO cut‐off levels: median UIC <150 μg/L representing inadequate iodine intakes, median UIC 150–249 μg/L representing adequate iodine intakes, and UIC ≥500 μg/L representing excessive iodine intakes.

Statistics in the texts are median (Q1, Q3) or geometric mean (95% confidence interval). We previously reported (Klevor et al., 2016) that women's adherence to supplement intake during pregnancy (i.e., percentage of follow‐up days women self‐reportedly consumed the supplements) was 88.1% for the IFA group; 87.0% for the MMN group; and 83.7% for the LNS group (Klevor et al., 2016).

## 3 RESULTS

The data presented here were collected between October 2010 and June 2012. The background characteristics of women in the subsample whose spot UICs were analysed are shown in Table 1. These characteristics were generally balanced across the three groups. There were no differences in these background characteristics between women in the subsample and those who were not selected for the UIC analysis (results not shown), except that the former had greater mean ± *SD* years of formal education (7.8 ± 3.3 vs. 7.3 ± 3.6 years; *p* = .045) and a lower percentage of women with a positive rapid diagnostic test for malaria (6% vs. 10%; *p* = .036). The percentage of women who reported using iodized salt *usually* or *always* was 31% at baseline and 33% at 36 weeks of gestation. Otherwise, the majority of the women reported that they *never* used iodized salt, or they used it *sometimes*.

**Table 1:** Background characteristics at enrolment of women randomly selected for urinary iodine analysis out of women who participated in a randomized trial of micronutrient supplementation during pregnancy in a semiurban setting in Ghana, by group^a^

Characteristics	IFA (*n* = 94)	MMN (*n* = 102)	LNS (*n* = 101)
Age (years)	26.3 ± 5.2 [92]	26.0 ± 5.1 [102]	26.9 ± 5.6 [101]
Years of formal education (years)	7.8 ± 2.9 [92]	7.5 ± 3.2 [102]	8.1 ± 3.8 [101]
Weight (kg)	63.0 ± 11.6 [91]	62.8 ± 11.8 [100]	61.9 ± 9.2 [98]
Height (cm)	158 ± 5.0 [91]	159 ± 5.7 [100]	159 ± 5.2 [98]
MUAC (cm)	28.3 ± 4.0 [91]	27.8 ± 3.7 [100]	27.8 ± 3.4 [98]
Triceps skinfold (mm)	19.3 ± 7.1 [91]	18.5 ± 7.2 [100]	18.7 ± 6.3 [98]
Body mass index (kg/m^2^)	25.1 ± 4.2 [91]	24.8 ± 4.2 [100]	24.6 ± 3.6 [98]
HFIAS score^b^	2.5 ± 3.7 [92]	2.2 ± 3.3 [101]	2.1 ± 3.0 [101]
Gestational age at enrolment (weeks)	16.2 ± 3.0 [92]	16.1 ± 3.1 [102]	16.1 ± 3.2 [101]
Nulliparous women, *n*/total (%)	37/92 (40.2)	37/102 (36.3)	34/101 (33.7)
Hb <100 g/L, *n*/total (%)	9/92 (9.8)	19/102 (18.6)	10/101 (9.9)
Positive malarial RDT, *n*/total (%)	4/92 (4.3)	4/102 (3.9)	9/101 (8.9)

*Note*. HFIAS = Household Food Insecurity Access Scale; IFA = iron and folic acid; LNS = lipid‐based nutrient supplement; MMN = multiple micronutrients; RDT = rapid diagnostic test (Clearview Malarial Combo; Vision Biotech, which detected *Plasmodium falciparum* and non‐*P. falciparum* histidine‐rich protein 2); SQ‐LNS = small‐quantity lipid‐based nutrient supplements; Hb = haemoglobin.

a *n* = 295. IFA group: Women were assigned to receive 60 mg Fe + 400 mg folic acid per day during pregnancy; MMN group: Women were assigned to receive 18 vitamins and minerals (including 20 mg Fe) per day during pregnancy; LNS group: Women were assigned to receive 20 g SQ‐LNS per day with the same micronutrients as the MMN group + calcium, phosphorus, potassium, and magnesium as well as macronutrients during pregnancy. Values are means ± *SD*s (*n*) unless otherwise indicated. *n*/total indicates the number of participants whose response was “yes” for the variable in question per total number of participants analysed for the variable in question.

b HFIAS score is a proxy indicator for household food insecurity (Coates et al., 2007); higher values represent higher food insecurity.

Results of the analysis of the UICs of women, by group at baseline and 36 weeks of gestation, are presented in Table 2. At baseline, the median (Q1, Q3) UIC for the MMN group appeared lower than the overall median (137 [78, 221] μg/L). The median change in UIC from baseline to 36 weeks of gestation was positive for the MMN and LNS groups, whereas the reverse was true for the IFA group.

**Table 2:** UIC (μg/L) of women who received IFA, MMN, and SQ‐LNS during pregnancy in the iLiNS DYAD‐Ghana trial, by group^a^

	Group			
UIC (μg/L)	IFA (*n* = 91)	MMN (*n* = 100)	LNS (*n* = 101)	*p*
Baseline^b^	150 (94, 234)	119 (67, 203)	151 (88, 218)	
Change (enrolment to 36 weeks of gestation)	−24 (−123, 52)	39 (−34, 122)	14 (−64, 102)	
36 weeks of gestation^c^	116 (101, 138)^e^	161 (133, 184)^f^	158 (132, 185)^f^	.004
36 weeks of gestation^d^	113 (100, 138)^e^	162 (132, 182)^f^	159 (135, 186)^f^	.001

*Note*. UIC = urinary iodine concentration; iLiNS = International Lipid‐based Nutrient Supplements; IFA = iron and folic acid; LNS = lipid‐based nutrient supplements; MMN = multiple micronutrients; SQ‐LNS = small‐quantity lipid‐based nutrient supplements. Analysis at 36 weeks of gestation was based on analysis of covariance (SAS PROC GLIMMIX).

a *n* = 292. IFA group: Women were assigned to receive 60 mg Fe + 400 mg folic acid per day; MMN group: Women were assigned to receive 18 vitamins and minerals including 250 μg iodine per day; LNS group: Women were assigned to receive 20 g SQ‐LNS per day with the same micronutrients as the MMN group, plus calcium, phosphorus, potassium, and magnesium as well as macronutrients.

b Baseline values are median (Q1, Q3).

c Values at 36 weeks of gestation are geometric means (95% CI) obtained by back‐transforming the log‐mean UIC after controlling for baseline UIC, with Tukey adjustment for multiple comparison. Geometric mean (95% CI) values with different superscript letters are significant different.

d Values at 36 weeks of gestation are geometric means (95% CI) obtained by back‐transforming log‐mean UIC after controlling for baseline UIC as well as years of formal education, parity, season of enrolment, and household assets index, with Tukey adjustment for multiple comparison. Geometric mean (95% CI) values with different superscript letters are significant different.

At 36 weeks of gestation, controlling for baseline UIC, the MMN and LNS groups did not differ significantly in geometric mean UIC, but each of these groups had a geometric mean UIC that was significantly greater than that of the IFA group. These results remained unchanged after controlling for additional background variables. The geometric mean (approximately median) UICs of the MMN and LNS groups were each greater than 150 μg/L, the WHO cut‐off for adequate iodine intakes, whereas the value for the IFA group was less than 150 μg/L. None of the geometric means was close to being ≥500 μg/L, the WHO cut‐off reflecting excessive iodine intakes.

Of the eight potential effect modifiers tested, three (gestational age at enrolment, parity, and baseline UIC) had a significant interaction with the intervention group (Table 3). There was a significant difference between intervention groups in UIC at 36 weeks of gestation among women enrolled in either the first or second trimester, but the groups that differed varied: UIC was higher in those in the MMN versus IFA group among those enrolled in the first trimester, whereas it was higher in both the MMN and LNS groups, compared with IFA, among those enrolled in the second trimester. With regard to parity, intervention groups differed in UIC at 36 weeks among parous women, but not among nulliparous women. Last, the effect of the intervention group was more evident in those with baseline UIC <150 μg/L than in those with higher baseline UIC.

**Table 3:** Geometric mean UIC (μg/L) at 36 weeks of gestation of women who received IFA, MMN, and SQ‐LNS during pregnancy in the iLiNS DYAD‐Ghana trial, stratified by subgroups of gestational age at enrolment, parity, and baseline UIC^a^

Baseline characteristics and subgroups	IFA^b^	MMN^b^	LNS^b^	*p* c	*p* d
Gestational age at enrolment (weeks)				.022	
In first trimester (*n* = 44)	99 (71, 139)^e^	156 (109, 223)^f^	106 (73, 146)^e^		.021
In second trimester (*n* = 248)	122 (103, 146)^e^	157 (131, 187)^f^	171 (143, 203)^f^		.015
Parity				.035	
Nulliparous (*n* = 108)	117 (88, 147)	130 (98, 173)	117 (93, 153)		.74
Parous (*n* = 184)	109 (97, 149)^e^	185 (144, 212)^f^	187 (149, 222)^f^		.001
Baseline UIC				.021	
UIC ≥ 150 μg/L (*n* = 147)	140 (112, 175)	210 (164, 268)	161 (131, 198)		.059
UIC < 150 μg/L (*n* = 145)	100 (79, 126)^e^	132 (108, 161)^f^	156 (124, 198)^f^		.025

*Note*. IFA = iron and folic acid; LNS = lipid‐based nutrient supplement; MMN = multiple micronutrients; SQ‐LNS = small‐quantity lipid‐based nutrient supplements; UIC = urinary iodine concentration. Analysis at 36 weeks of gestation was based on analysis of covariance (SAS PROC GLIMMIX).

a *n* = 292. IFA group: Women were assigned to receive 60 mg Fe + 400 mg folic acid per day; MMN group: Women were assigned to receive 18 vitamins and minerals including 250 μg iodine per day; LNS group: Women were assigned to receive 20 g SQ‐LNS per day with the same micronutrients as the MMN group, plus calcium, phosphorus, potassium, and magnesium as well as macronutrients.

b Values are geometric means (95% CI), adjusted for variables significantly associated with the outcome variable at α < .1 in bivariate analysis: baseline UIC, years of formal education, parity, season of enrolment, and household assets index. Geometric mean (95% CI) values with different superscript letters are significant different.

c *p*‐values are for interaction with intervention group.

d *p*‐values compare all three groups in each stratum.

The interaction between reported use of iodized salt and intervention group was not significant. However, the likelihood that median UIC would fall below the WHO cut‐off for adequacy differed depending on the reported use of iodized salt. At baseline, median UIC was 178 μg/L in women who reported using iodized salt *usually* or *always* at either baseline or 36 weeks of gestation and 126 μg/L for women who did not report using iodized salt *usually* or *always* at either time point; within each of these categories of women, the median UICs of the three intervention groups were comparable at baseline. At 36 weeks of gestation, median UIC among women who reported using iodized salt *usually* or *always* at either time point was 188 μg/L in the IFA group, 228 μg/L in the MMN group, and 211 μg/L in the LNS group; among women who did not report using iodized salt *usually* or *always* at either time point, median UIC was 86 μg/L in the IFA group, 137 μg/L in the MMN group, and 133 μg/L in the LNS group.

## 4 DISCUSSION

We found that in this semiurban setting in Ghana, the consumption of SQ‐LNS or MMN, compared with IFA, during pregnancy increased maternal UIC. At 36 weeks of gestation, the iodine status of women provided with MMN or SQ‐LNS was adequate according to WHO‐recommended cut‐off levels, whereas the iodine status of women provided with IFA was insufficient. The impact of MMN or SQ‐LNS consumption during pregnancy on UIC appeared to be greater in parous than in nulliparous women.

We previously mentioned several strengths, as well as weaknesses, of our study (Adu‐Afarwuah et al., 2015; Adu‐Afarwuah et al., 2017). The former include use of a fully randomized design and blinding of laboratory analysts involved in the analysis of UIC. We paid special attention to ensuring data quality. The study weaknesses included our inability to fully blind all nonlaboratory study staff and participants to the supplementation allocation because of the obvious differences between the IFA and MMN capsules compared with the SQ‐LNS sachets.

The WHO recommends the use of median UIC from spot urine samples to describe iodine status in settings where salt iodization programmes are in place (WHO, 2007). Median UICs from spot urine samples (WHO, 2007) and the cut‐offs (WHO, 2013) are recommended for assessing iodine intakes in populations (WHO, 2013). In individuals, UICs are known to vary (between and within days) considerably (Rasmussen, Ovesen, & Christiansen, 1999; WHO, 2007). We did not examine the proportion of individuals within versus outside of the adequate range for UIC because we had no data to estimate and correct for day‐to‐day intraindividual variability.

Several previous trials have compared women given MMN including iodine with those given IFA during pregnancy, including studies in Pakistan (Bhutta et al., 2009), Guinea Bissau (Kaestel, Michaelsen, Aaby, & Friis, 2005), Nepal (Osrin et al., 2005), Burkina Faso (Roberfroid et al., 2008), Indonesia (Sunawang, Utomo, Hidayat, Kusharisupeni, & Subarkah, 2009; Supplementation with Multiple Micronutrients Intervention Trial Study et al., 2008), Bangladesh (Eneroth et al., 2010), Niger (Zagre, Desplats, Adou, Mamadoultaibou, & Aguayo, 2007), and China (Zeng et al., 2008), but we found only one study that reported UIC: In Indonesia (Sunawang et al., 2009), the investigators reported that “iodine levels increased” in pregnant women receiving either the MMN supplement or IFA. It is unclear what the magnitude of the increase was and what explained the increase in UIC in the group receiving IFA. In the Rang‐Din Nutrition Study (Mridha et al., 2017), the geometric mean UIC of the Bangladeshi women (48 μg/L in early pregnancy and 29 μg/L at 36 weeks of gestation) was very low compared with that of the women in Ghana, and there was no significant effect of SQ‐LNS containing iodine on UIC. The investigators speculated that under such circumstances, the supplemental iodine was utilized in the thyroid gland, as opposed to being excreted in the urine.

Although there are no universally accepted cut‐offs for defining severe, moderate, or mild iodine deficiency in pregnant women based on UICs, the observed UIC of women in the IFA group at 36 gestational weeks may suggest iodine deficiency (Zimmermann, 2007), particularly for those who did not report using iodized salt usually or always. These results for the IFA group are comparable with those from several iodine‐deficient countries, in which median UICs during pregnancy have been found to be relatively low (Zimmermann, 2007). In contrast, the results for the MMN and LNS groups are similar to those of longitudinal and cross‐sectional studies from several iodine‐sufficient countries (where all salt is iodized, or dietary iodine comes from either iodized salt or other food sources) in which the median UICs during pregnancy were ≥140 μg/L (Zimmermann, 2007).

Median UIC of pregnant women recently reported for Ghana (Iodine Global Network, 2017), which was based on a nationally representative survey, suggests that at the national population level, the iodine intakes of pregnant women are adequate. Our finding of inadequate iodine intakes, based on UIC, among women in the IFA group conflicts with this national report. It may be that the national level UIC results (Iodine Global Network, 2017) obscure regional or subregional disparities, which is common in such national cross‐sectional surveys (Doggui, El Ati‐Hellal, Traissac, Lahmar, & El Ati, 2016). The study site is not a known iodine deficiency‐endemic area, but much of the staple food consumed in the area comes from a nearby mountainous district (Upper Manya Krobo District) and may have low iodine content because of leaching from soil erosion (Zimmermann, 2015). Consequently, the pregnant women in the study area, who consume these staple foods, may have low iodine status (Zimmermann, 2015).

The low geometric mean UIC of women in the IFA group, whose supplement contained no iodine, may not be surprising. Since the mid‐1990s, the national salt iodization programme in Ghana has been the main driving force preventing IDD in the country, and a considerable progress has been made in reducing IDD. The implementation of the programme has, however, been hampered by various challenges, including the difficulty in acquiring the needed fortificant (potassium iodate) and the large number of small‐scale salt producers, some of whom have little or no capacity to iodize the salt they produce (Nyumuah et al., 2012). The large percentage of households consuming inadequately iodized salt observed in previous national surveys (GSS, 2011, GSS et al., 2015), and supported by findings from this study, suggests that those challenges remain to some degree, despite attempts at resolving them (Nyumuah et al., 2012), and as a result, much of the salt produced in Ghana is still not iodized. Women know that noniodized salt is cheaper and more accessible, compared with the usually factory‐processed, fine‐textured, and branded iodized salt (Agbozo, Dera, Gloverb, & Ellahi, 2016; Avinash & Prabha Adhikari, 2002; Knowles et al., 2017).

Thus, our results suggest that without iodine supplementation, it is difficult for pregnant women in our study setting to achieve adequate iodine status, unless they use iodized salt *usually* or *always* during pregnancy. In fact, there are similar reports from other countries showing low iodine status of pregnant women even when a national salt iodization programme is in place (Mao et al., 2015; Oral et al., 2016). We did not measure the iodine status of infants born to women in our study, but in Morocco (Stinca et al., 2017) the iodine status (as measured by total thyroxine) of infants whose mothers were iodine deficient appeared to be worse than that of the women themselves. Although the full impact of mild iodine deficiency during pregnancy on fetal development may not be known, the consequences could include increased risk of lower verbal intelligence quotient later in life (Bath, Steer, Golding, Emmett, & Rayman, 2013; Stinca et al., 2017). Maternal serum thyroid hormone concentration or production may, however, be normal even when mean UIC is as low as 33 μg/L (Cao et al., 1994; Caron et al., 1997; Pedersen et al., 1993; Silva & Silva, 1981), although it is not certain what else may be affected when UIC falls below the 150 μg/L WHO cut‐off.

Our results also suggest that ensuring adequate iodine status of pregnant women in Ghana requires a policy for regular monitoring of the iodine status of the population, especially pregnant women, in addition to periodically determining the coverage of iodized salt consumption at the household level (GSS et al., 2015, GSS, 2011). Currently, the percentage of Ghanaian pregnant women showing obvious signs of iodine deficiency is low (Government of Ghana, 2009), but a large number of women living in certain parts of the country may be consuming insufficient iodine.

Results from subgroup analyses in randomized controlled trials should be interpreted with caution (Brookes et al., 2001; Sedgwick, 2014); however, our subgroup analyses were prespecified (Brookes et al., 2001; Moher et al., 2010; Sedgwick, 2014), and our finding that MMN and SQ‐LNS consumption did not affect the UIC of nulliparous women is presented to stimulate further investigation, as it may reflect the greater vulnerability of nulliparous women compared with their parous counterparts. As reported previously (Adu‐Afarwuah et al., 2015), apart from being younger, the first‐time mothers weighed less, had a lower mean haemoglobin concentration, and were more likely to be anaemic or test positive for malaria at baseline than parous women. It is possible that some of these conditions, for example, increased risk of anaemia, could have negatively affected the impact of MMN and SQ‐LNS supplementation. In the Cote d'Ivoire (Zimmermann, Adou, Torresani, Zeder, & Hurrell, 2000), iron deficiency anaemia weakened the efficacy of iodine supplementation in goitrous children possibly by reducing the activity of iron‐containing thyroid peroxidase (Hess, Zimmermann, Arnold, Langhans, & Hurrell, 2002), which can impair thyroid hormone production. The increased risk of anaemia and malaria in nulliparous women also could have reduced iodine intakes through the reduction of food consumption because of anorexia or the cultural practice of withdrawal from food during illness (Scrimshaw, 1992).

We conclude that in this semiurban setting in Ghana, the iodine intakes of pregnant women appear to be insufficient despite the national salt iodization programme and that the consumption of SQ‐LNS or MMN supplements providing iodine at the current WHO‐recommended daily dose increases the likelihood of adequate iodine status. Although universal salt iodization remains to be achieved in Ghana, supplementation with SQ‐LNS or MMN would ensure optimal iodine status among pregnant women in this setting.

## CONFLICTS OF INTEREST

S. Adu‐Afarwuah, R. T. Young, A. Lartey, H. Okronipa, P. Ashorn, U. Ashorn, and K. G. Dewey declare no conflicts of interest. At the time of the study, M. Zeilani was an employee of Nutriset S.A.S., which is a commercial producer of LNS products. The funder of the study had no role in the study design; data collection, analysis, and interpretation; and the preparation of the manuscript.

## CONTRIBUTIONS

SA‐A, AL, PA, MZ, and KGD designed the research; MZ was responsible for the development and production of the SQ‐LNS used in the study based on the specifications agreed upon by the iLiNS Project; SA‐A, AL, and HO conducted the research; SA‐A and RTY performed the statistical analysis; SA‐A and KGD wrote the manuscript; and AL, HO, PA, UA, and MZ reviewed the draft manuscript. All authors read and approved the final manuscript.
